# Meet up‐and‐coming analytical scientists – Fabio Pereira Gomes

**DOI:** 10.1002/ansa.202200042

**Published:** 2022-12-02

**Authors:** Fabio Pereira Gomes

**Affiliations:** ^1^ Department of Molecular Medicine Scripps Research La Jolla California USA


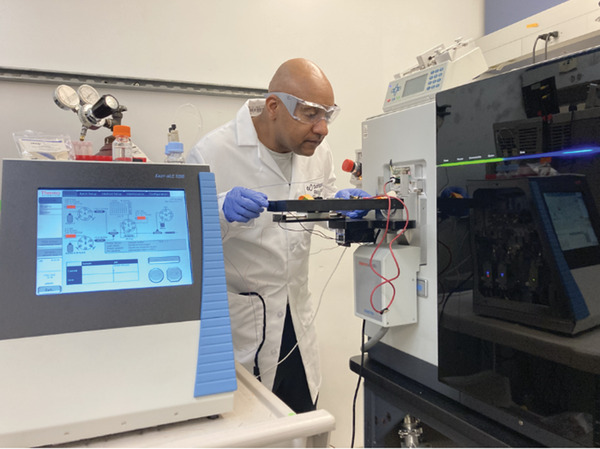
Analytical sciences are among the most dynamically developing fields and have been inherently integrated into many various scientific disciplines. At the same time, early career researchers (ECRs) are among those whose contribution to this dynamic growth cannot be simply overestimated. Hence, in this special issue “From one ECR to the next”, we are presenting a series of editorials with questions and answers from five emerging scientists from different analytical fields including omics, environmental and data sciences. Importantly, all our guests boast not only scientific excellence and high‐quality research but also the substantial international experience gained during their PhD or postdoctoral training. For this editorial, we are presenting Dr. Fabio P. Gomes.

Dr. Gomes is a Postdoctoral Researcher at the Scripps Research. He holds a Ph.D. in Analytical Chemistry from the University of Queensland. His research interests include the use of innovative mass spectrometry (MS)‐based methods to investigate the structure‐function relationship of intact proteoforms and their complexoforms withincells. He is currently developing and applying MS approaches to structurally elucidate intact complexoforms (protein complexes formed by intact monomeric proteoform arrangements) in breast cancer cells, and to interrogate intact proteoforms in single cardiomyocyte cells. Proteins adopt multiple proteoforms as a result of various structural changes (e.g. posttranslational modifications [PTMs] and truncations).

## What is your original background?

I am an Afro‐Brazilian citizen and US permanent resident. I was born and raised in Sao Paulo (Brazil), where I completed both my undergraduate degree and a master's degree with graduate research in Pharmaceutical Sciences. I then moved to the University of Queensland (Australia) to complete my PhD in Analytical Chemistry. After graduation, I moved to the United States where I have held postdoctoral positions in two laboratories led by world leader proteomics: first with Dr. Catherine Fenselau at the University of Maryland (2017–2018) and currently with Dr. John R. Yates III at the Scripps Research (2018–present).

## What is your current research focus?

I have 2 overaching research goals. My first goal is to develop and improve technologies for probing intact proteoforms and their complexoforms within the intracellular space. My second goal is to apply these technologies to better understand the molecular mechanisms that govern metastatic tumors, drug resistance, and how lipids bind and modulate the biological activities of important drug targets such as membrane proteins. I am excited about the native top‐down proteomics (nTDP) strategy I developed to interrogate complexoforms in breast cancer cells. I plan to extend this approach to investigate the hypothesis that the biological actions of estrogen and antiestrogen drugs in the development of metastatic breast tumors and drug resistance are regulated by estrogen receptor alpha (ER‐alpha) proteoforms and complexoforms. I am also enthusiastic about the top‐down proteomics (TDP) strategy I developed to interrogate intact proteoforms in single cardiomyocyte cells. I plan to extend this approach to capture the breast cancer cell‐to‐cell heterogeinety. I will soon be submitting two first‐author manuscripts for publication that are related to these two efforts.

## What is your biggest motivation to work in analytical science?

Discovery is my major motivation. Analytical Science is a fascinating branch of chemistry that allows scientists to gain insights into the structure and properties of biological systems by better understanding complex biological processes at the molecular level. This information can then be used to develop effective therapeutic interventions. For example, proteoforms and their complexoforms in biologically relevant samples can be separated by chromatography or electrophoresis, and then powerful analytical techniques such as MS can be used to precisely identify and quantify these macromolecules and other biomolecules in the eluates.

## Of all your research projects, which one was your favourite and why?

As mentioned previously, I am currently developing TDP‐based methods to test the hypothesis that the biological actions of estrogens and antiestrogen drugs in the development of breast cancer and resistance to endocrine therapy are regulated by ER‐alpha proteoforms and complexoforms. I want to define how different receptor proteoforms and complexoforms lead to specific signaling outcomes and how these are impacted by endocrine treatments and growth factor‐mediated resistance. I am particularly excited about this work because I strongly believe it will have a very high impact on many fields, including estrogen receptor biology, basic mechanisms of transcription and the study of proteoforms more generally, which will significantly impact the understanding of resistance to endocrine therapy as well as cancer signalling and heterogeneity.

## What was your motivation for choosing postdoctoral training?

Over the course of my PhD, I became very interested in protein MS. Most cellular functions, including those linked to pathological and physiological states, are performed by protein complexes that often assemble via non‐covalent interactions of monomeric protein subunits. As mentioned previously, proteins adopt multiple proteoforms as a result of various structural changes (e.g. PTMs and truncations). Proteoforms influence the formation, stability and activity of functional protein complexes, and can form numerous functional “complexoforms” from a single protein complex. Individual proteoforms can independently modulate numerous biological processes, and they may also serve as important markers of disease. MS allows scientists to understand the molecular basis of physiological and pathological processes and to identify novel drug targets that can ultimately help patients. Thus, for my postdoctoral training, I decided to pursue research that uses MS‐based methods and biological techniques to elucidate the structure of proteoforms and their complexoforms in the intracellular space.

## What was your biggest (if any) culture shock experience in the country of your postdoc?

Brazil is largely influenced by American culture. For instance, several people in Brazil celebrate Thanksgiving and have Halloween parties. In addition, American music and movies are predominant in many parts of Brazil, so I was aware of many things about American culture before moving to the United States. While I continue to admire American culture, I was surprised by the underrepresentation of black scientists in academic and industrial settings. I now understand that this is a global issue; I have observed racial disparities in all the countries I lived in, including Brazil, Australia and Germany. Sadly, there are still many institutional, social and emotional barriers that impede talented black children from becoming scientists.

## In your scientific career, what was the best or worst advice you ever heard from anyone?

As a graduate student and postdoctoral researcher, I have been fortunate to benefit from supportive mentors and colleagues who provided/provide me with useful advice for building a career as a successful independent scientist. Among the many important pieces of advice, I would say that “Be courageous, innovative, collaborative and respectful, as well as learn from your mistakes as they are necessary to make you a better colleague/scientist and to get the right answers” might be the best.

## What advice would you give to someone looking for a postdoc position now?

Make sure you know what you want for your future career and then choose a lab that can help you to achieve your career goal. On top of your strong desire and motivation to succeed, a supportive postdoctoral adviser and a productive and collaborative environment can be key to your career growth. Learn as much as possible from colleagues, mentors and collaborators. Publish quality papers and make sure the quality of the paper is the same for high‐impact or low‐impact factor journals. Write papers, reviews and grants, even if you will not pursue an academic career. I believe that the ability to communicate effectively (speaking and writing) will open doors and make a significant difference in your career in either industry or academic settings. Work with your heart, be kind and friendly and listen to advise but make your decisions.

## What is your favourite non‐scientific activity?

I enjoy many different things, such as listening to music, watching movies, swimming, going out with my wife, spending time with my friends, playing soccer and having a good beer. I also enjoy calling my mother and niece in Brazil. Getting together for barbecues is also fun. My wife and I make barbecues all the time.

## Who (three people but not scientists!) would you invite to a dream dinner party?

There are many people I would like to invite to my dream dinner party, including my mother, niece and wife, but I would also love to invite Serena Williams, Barack Obama and Michael Jordan. As an international tennis legend, Serena Williams has been a role model for black children worldwide, especially girls from underprivileged communities. Barack Obama's landmark election as the first Afro‐American president of the United States was a transformative event for black people across the globe, giving them hope that everything is possible with hard work and education. I would include Michael Jordan because of the profound effect his magic moves had on me as a young fan and on the sport of basketball. Michael Jordan is still a source of inspiration for many people and will be remembered as the greatest or one of the greatest basketball players ever. His post‐basketball career success would make him an even more interesting dinner guest.

## CONFLICT OF INTEREST

The author declares no conflict of interest.

